# Initiating a regenerative response; cellular and molecular features of wound healing in the cnidarian *Nematostella vectensis*

**DOI:** 10.1186/1741-7007-12-24

**Published:** 2014-03-26

**Authors:** Timothy Q DuBuc, Nikki Traylor-Knowles, Mark Q Martindale

**Affiliations:** 1 University of Hawaii, Kewalo Marine Laboratory, 41 Ahui Street, Honolulu, HI 96813, Hawaii; 2 University of Florida, Whitney Marine Laboratory, 9505 Oceanshore Boulevard, St. Augustine, FL 32080, USA; 3 Stanford University, Hopkins Marine Station, 120 Oceanview Blvd., Pacific Grove, CA 93950, USA

**Keywords:** Cnidarians, ERK signaling, Gastrulation, Glycoprotein, Lateral gene transfer, Metalloproteinase, Microarray, *Nematostella vectensis*, Regeneration, Wound healing

## Abstract

**Background:**

Wound healing is the first stage of a series of cellular events that are necessary to initiate a regenerative response. Defective wound healing can block regeneration even in animals with a high regenerative capacity. Understanding how signals generated during wound healing promote regeneration of lost structures is highly important, considering that virtually all animals have the ability to heal but many lack the ability to regenerate missing structures. Cnidarians are the phylogenetic sister taxa to bilaterians and are highly regenerative animals. To gain a greater understanding of how early animals generate a regenerative response, we examined the cellular and molecular components involved during wound healing in the anthozoan cnidarian *Nematostella vectensis*.

**Results:**

Pharmacological inhibition of extracellular signal-regulated kinases (ERK) signaling blocks regeneration and wound healing in Nematostella. We characterized early and late wound healing events through genome-wide microarray analysis, quantitative PCR, and *in situ* hybridization to identify potential wound healing targets. We identified a number of genes directly related to the wound healing response in other animals (metalloproteinases, growth factors, transcription factors) and suggest that glycoproteins (mucins and uromodulin) play a key role in early wound healing events. This study also identified a novel cnidarian-specific gene, for a thiamine biosynthesis enzyme (vitamin B synthesis), that may have been incorporated into the genome by lateral gene transfer from bacteria and now functions during wound healing. Lastly, we suggest that ERK signaling is a shared element of the early wound response for animals with a high regenerative capacity.

**Conclusions:**

This research describes the temporal events involved during *Nematostella* wound healing, and provides a foundation for comparative analysis with other regenerative and non-regenerative species. We have shown that the same genes that heal puncture wounds are also activated after oral-aboral bisection, indicating a clear link with the initiation of regenerative healing. This study demonstrates the strength of using a forward approach (microarray) to characterize a developmental phenomenon (wound healing) at a phylogenetically important crossroad of animal evolution (cnidarian-bilaterian ancestor). Accumulation of data on the early wound healing events across numerous systems may provide clues as to why some animals have limited regenerative abilities.

## Background

Wound healing is the process of cellular contraction, movement and re-adhesion immediately after injury and is the precursor to regeneration of lost structures. During these cellular dynamics, other components such as immunity, cell death or proliferation, and nervous system inputs all interact during the process of scar-free healing [1]. Among animals with high regenerative capabilities, a unifying theme has emerged suggesting that the cells that re-epithelialize the wound provide the signals necessary to initiate regeneration [2]. Defects in the wound healing program, including excessive scar formation or mechanically manipulating the wound, can block regeneration, even in animals with a high regenerative capacity [3-5]. Understanding the process of wound closure in diverse animal groups that vary in regenerative capacity may help reveal factors correlated with the loss of regeneration.

**Figure 1:**
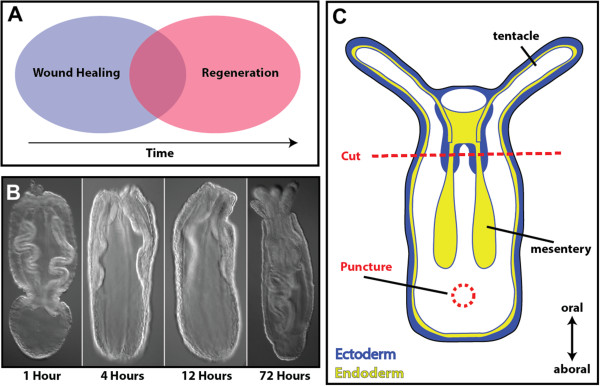
**Wound healing, a necessary precursor to regeneration. (A)** Wound healing and regeneration are separable developmental processes that may involve different gene cascades. **(B)** Head regeneration is a rapid process in *Nematostella* where structural integrity is renewed approximately 72 hours after injury. **(C)** Schematic representation of the experimental setup of regeneration and wound healing experiments using *Nematostella* juveniles. In regeneration experiments, animals are cut just below the pharynx region (red line). In wound healing experiments, animals are punctured at the central position between the mesenteries and the aboral pole. Puncture wounds span both sides of the animal creating two similar wounds.

The process of wound healing exists widely throughout the animal kingdom, yet after a significant loss of tissue, few animal groups can faithfully regenerate the entire complement of original tissue. The cnidarians (for example, corals, jellyfish, sea anemones) are diploblastic animals, consisting of ectodermal and endodermal tissue [6]. Many studies have demonstrated that cnidarians are a powerful model for understanding the evolution of bilaterians, because of their phylogenetic position (sister to bilaterians) and because they are more similar in terms of genomic content and organization to deuterostomes than other model systems [7-11]. As adults, cnidarians exhibit a high regenerative capacity with few limitations [12]. The medusazoan model system, *Hydra*, has long been a comparative model for regenerative study, bridging the gap between early animals, planarians, flies and vertebrates. The anthozoan cnidarian, *Nematostella vectensis*, is widely known as a comparative system for embryological studies, yet following bisection through their major longitudinal axis (the oral-aboral axis) both halves can regenerate into normal animals [13-18]. Unlike *Hydra*, anthozoans like *Nematostella* do not appear to have an I-cell population of precursor stem cells [19]. Instead, cell proliferation is required for the completion of the regenerative process in *Nematostella* and is first active 18 hours after injury [16,19,20]. Interestingly, regeneration experiments where wound healing was allowed to proceed but cellular proliferation was chemically blocked can be rescued by re-injuring the same untreated tissue, triggering mitosis and regeneration [16]. This suggests that wound healing acts as an initiator of regeneration in *Nematostella,* where the onset of proliferation may serve as an important transition between wound healing and a regenerative response (Figure 1A). Regeneration of lost oral structures takes approximately 72 hours (Figure 1B), yet little is known about the timing and transition from wound healing to regeneration in *Nematostella*. A genomic survey of stress response genes suggests that homologs of many vertebrate genes previously associated with wound healing are also present in the *Nematostella* genome, although a quantitative assessment of gene expression is lacking [21]. Overall, the high regenerative capacity and key phylogenetic position of cnidarians provide a unique opportunity to study the basic mechanism underlying animal wound repair. This type of study is of great interest in comparison to other highly regenerative animals (to see similarities) and in comparison to animals that lack the capacity to regenerate (to see differences). Studying new systems with forward approaches also provides unique opportunities for gene discovery.

The mitogen-activated protein kinase (MAPK) signaling pathway is present in all eukaryotic genomes and functions in a wide range of cellular processes including immune system regulation, proliferation, apoptosis, cell signaling and movement. A subset of the pathway, ERK signaling, regulates initial events of *Drosophila* wound closure by regulating actin dynamics around the site of injury [22]. In vertebrate cell culture, scratch assays show that ERK is localized in contractile cells around the wound margin [23]. ERK signaling is also a key regulator of the *Grainyhead* gene family, a group of genes known for their role in establishment of the epithelial layer and their role in wound healing across animals [24]. Activation of ERK is also linked with the innate immune response in a number of animals [25-27] where MAPK signaling is likely the main signaling system for host-parasite or symbiont-host interactions. Among the results reported here, we found that the inhibition of ERK signaling blocked both wound healing and regeneration in *Nematostella*. Using a diverse set of approaches we describe many of the components involved during wound healing in *Nematostella*, and show that a universal set of genes are activated during different types of wound healing prior to regeneration.

## Results

### Cellular events orchestrated during wound healing of *Nematostella vectensis*

**Figure 2:**
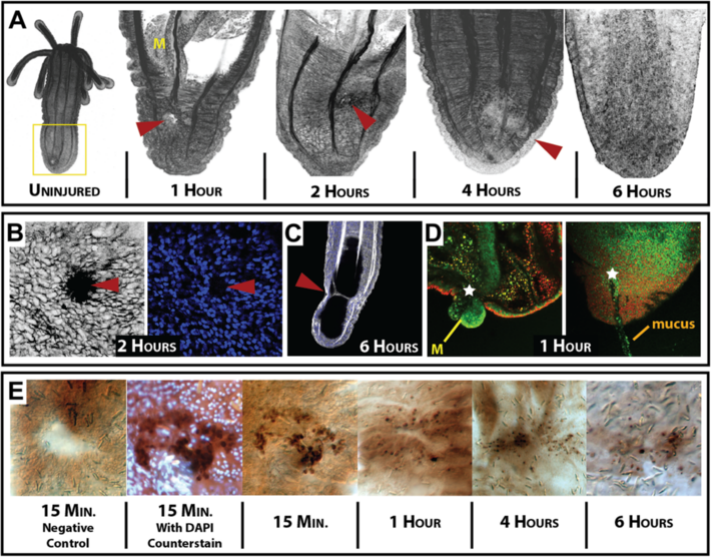
**Biological events during *Nematostella *wound healing. (A)** Timeline of morphological events during the first six hours after injury. Filamentous actin, a core component of the extracellular matrix and muscle fibers, was labeled with Phallacidin-FL (false-colored black). A comparison of uninjured animals (far left), to injured animals shows that after six hours (far right), the wound is unidentifiable. The yellow box around the aboral side of the uninjured animal designates the zone of injury throughout our study. At one hour, the animal exhibits a deflated collapsed state due to water loss from the gastrovascular cavity, and the mesentery can be seen extending towards the wound (yellow M). **(B)** By hour two, high concentrations of actin are found in the cells along the margin of the wound (left), with nuclei positioned outside the wounded area (right), suggesting these are actin filopodial projections into the wounded area. **(C)** In a small percentage of animals, long actin filopodia can be found connecting the two parallel wounds. **(D)** Punctured live animals (stained with acridine orange) often plug their wound with mesentery structures (left) and secrete a mucus-like material from the injury site (right). **(E)** Time series of potential apoptotic cells during puncture wound healing, as revealed by the DeadEnd tunel assay (labels cells with DNA damage). Control samples show no signal immediately after injury; the highest concentration of signal is seen immediately (15 minutes) after injury and the signal diminishes over time. (Negative control did not have recombinant terminal deoxynucleotidyl transferase enzyme - as specified from the protocol). Counterstaining with DAPI (image 2) designates that the kit is identifying cells with extensive DNA damage (apoptotic). (All red arrow heads or white stars indicate the position of the injury site).

Punctures were formed by passing a glass needle through ectodermal and endodermal layers of the aboral portion of animals (Figure 1C) and took approximately six hours to heal (Figure 2A). Immediately after injury, tissue in the aboral portion of the animal became deflated. This was primarily due to the loss of water within the gastrovascular system, and the inability to stop water-flow from exiting the wound. This compacted form lasted approximately four hours, until animals were capable of holding water again (Additional file 1). At two hours after injury (Figure 2B, left), an enrichment of actin was seen around the injury site, but a hole was still visible that did not contain nuclei (Figure 2B, right). This suggests that cells immediately surrounding the injury site stretch actin filopodia towards the central portion of the wound and create connections to pull the wound closed. A small subset of animals was found to make long actin strings that stretched from one side of the animal to the opposite side (Figure 2C). This is likely due to the tissue having injuries on both sides and cells on each side stretching to fill the wound, while potentially coming in contact with one another. Overall, scar-free wound healing finished approximately six to eight hours after injury, unless substantial damage to retractor muscles inadvertently occurred during injury. These structures were not repaired at six hours and may need proliferation to regrow [16,28].

During the early stages of wound healing, mesentery structures plugged the wound for short periods of time (Figure 2D, left; Additional files 2 and 3). This behavior could provide signals from the endoderm to the outer ectoderm that an injury has occurred. A similar phenomenon was found during regeneration, if the aboral regenerate retained mesentery tissue after bisection (data not shown). Interestingly, a sticky mucus-like material was excreted from the wound (Figure 2D, right; Additional file 4) that contained cellular debris. We used Alcian blue and Periodic acid-Schiff’s reagent to identify potential areas of mucin production within *Nematostella*. We found potential regions of mucus synthesis and secretion along the pharynx (Additional file 5A), the outer epithelium (Additional file 5B) and the base of the mesenteries (Additional file 5C), whereas little mucus was visible along the tentacles (Additional file 5D). When we looked at mucus production during regeneration, we found that wild-type animals appear to have a greater amount of mucus along the endodermal tissue compared to U0126 animals (Additional file 5E-E’). At one hour after injury, mucus was found throughout tissue along the site of injury, with little staining in U0126 animals (Additional file 5F-F’). By four hours, extensive mucus staining was visible within the endoderm of wild-type animals and appeared less abundant in U0126 animals (Additional file 5G-G’). At 12 hours, mucus staining appeared diminished in both controls and U0126-treated animals, with slightly elevated levels in U0126 samples (Additional file 5H-H’). We did not identify any areas enriched with Alcian blue staining, except the tips of the tentacles (data not shown).

Apoptosis initiates head regeneration in *Hydra*, and wounding along the body column can create new buds [29]. In *Nematostella*, we found that apoptosis is also activated upon injury along the ectodermal surface (Figure 2E). Expression of apoptotic signal appears greatest minutes after injury but a small amount can be found as late as six hours after injury. We quantified the number of DAB positive cells around the site of injury compared to the total number of nuclei within close proximity of the wound (Additional file 6A). A ratio of tunel positive cells/total nuclei suggests immediately after injury there is eight times the number of cells undergoing apoptosis than in uninjured animals (Additional file 6B). This ratio ranges from 16% immediately after injury to 9% after six hours.

### MAPK (ERK) signaling is crucial for proper wound healing

Previous studies suggest a link between activation of apoptosis and the onset of proliferation during regeneration in vertebrates and invertebrates alike [30]. Apoptosis is driven through a number of MAPK signaling components and has known roles in *Hydra* regeneration without affecting wound healing [29]. We chose to conduct a pharmacological inhibitor screen to see what other signaling pathways have a potential role in wound healing and regeneration. During regeneration, the wnt-signaling pathway is suggested to play a key role in axis formation in both *Hydra* and *Nematostella*[15,29]. Inhibitors for notch, transforming growth factor beta (TGFβ), and MAPK were tested to determine if we could differentially affect wound healing or regeneration.

Inhibition of notch signaling using the λ-secretase inhibitor, N-[N-(3,5-Difluorophenacetyl)-L-alanyl]-S-phenylglycine t-butyl ester (DAPT), blocks head reformation (Additional file 7) with no morphological changes in wound healing. TGFβ signaling is one of the first pathways activated and a known regulator of vertebrate wound healing [31]. Surprisingly, we did not see any phenotypic change in wound healing or regeneration with the TGFβ inhibitor SB431542. Neither inhibitor was analyzed further because they did not exhibit morphological changes to wounded animals.

**Figure 3:**
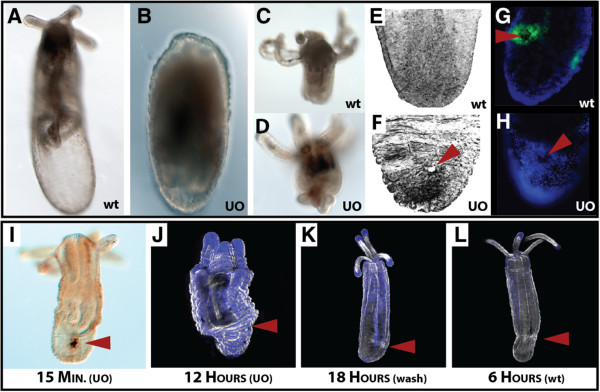
***Nematostella *wound healing is mediated by ERK signaling.** Inhibition of ERK signaling by U0126 disrupts normal wound healing and regeneration. **(A)** Animals that underwent oral-aboral bisection regenerate oral structures by 72 hours, whereas **(B)** animals soaked for three days in 10 μM U0126 (MAPK inhibitor) did not regenerate lost heads. **(C,D)** Heads that had to regenerate lost aboral structures exhibited wound healing defects when soaked in 10 μM U0126 for three days. **(E,F)** Aboral view of phallacidin-stained animal tissue exposed to 10 μM U0126 for six hours after injury lacked proper wound closure. **(G,H)** Puncture wounds caused local activation of phosphorylated-ERK around the site of injury, where incubation with U0126 blocks phosphorylated activity of ERK around the site of injury. **(I)** Animals were pre-soaked in U0126 for one hour, injured, and analyzed with the DeadEnd tunnel assay for apoptosis. Exposure of injured animals to U0126 did not block the activation of apoptosis after injury. **(J-L)** Scanning laser confocal images of **(J)** injured animals that were soaked in U0126 for 12 hours; **(K)** injured animals that were soaked in U0126 for 12 hours before the U0126 removed, and then placed in 1/3× seawater for an additional six hours; and **(L)** wild-type injured animals at six hours. Animals exposed to U0126 for prolonged periods of time **(J)** remained in a compacted state as a result of wounds never healing. Removal of U0126 at 12 hours allowed animals to reinitiate wound healing **(K)** and the animals were morphologically similar to animals at six hours **(L)**. (Red arrowhead in all images is used to identify the position of the wound). Black color in **E** and **F** and the white color in **J** to **L** are from phallacidin-FL, while the blue color in **G** and **H** and **J** to **L** is created from DAPI-labeled nuclei. wt, wild-type; UO, U0126.

The MAPK inhibitor of ERK signaling (U0126) blocked regeneration with dramatic wound healing defects in both head regeneration (Figure 3A,B) and aboral regeneration (Figure 3C,D). In aboral regenerates, the site of injury appeared wrinkled with the wound opening still visible (data not shown) whereas oral regenerates often had excess tissue that did not reintegrate into the animal (Figure 3D). Interestingly, the loss of regenerative ability occurred in animals that were exposed to U0126 prior to injury (Figure 3A,D) and when drug was added at eight hours after injury (data not shown). This suggests that ERK signaling may have multiple functions spanning the whole process of wound healing and regeneration. We set forth to determine the role of ERK signaling during wound healing because it was the only pathway that caused wound healing defects after injury.

Using our puncture assay methodology, we found that inhibition of ERK signaling by U0126 caused aboral puncture wounds to remain open after six hours, the normal time for wounds to heal (Figure 3E,F) and eliminated local phosphorylation of ERK at one hour after injury (Figure 3G,H). Incubation of animals in U0126 did not result in a loss of apoptotic signal immediately after injury (Figure 3I), suggesting that apoptosis alone cannot initiate regeneration. Long-term exposure to U0126 during wound healing resulted in animals remaining in a compressed state (Figure 3J). Washing out U0126 after 12 hours was sufficient to reinitiate wound healing (Figure 3K), and these animals appeared to be morphologically similar to wild-type animals six hours after injury (Figure 3L). These data show that U0126 is capable of blocking wound healing, without affecting the normal apoptotic program. This suggests that cellular movement and adhesion could be primary targets of ERK signaling, because proliferation is not active until much later during regeneration [16]. In fact, U0126 -treated animals did not show large amounts of actin around the site of injury (Figure 3F) as seen in Figure 2C. Overall, inhibition of ERK signaling by U0126 is a reversible process that is necessary for *Nematostella* wound closure.

### Transcriptional component of wound healing as revealed by microarray

**Figure 4:**
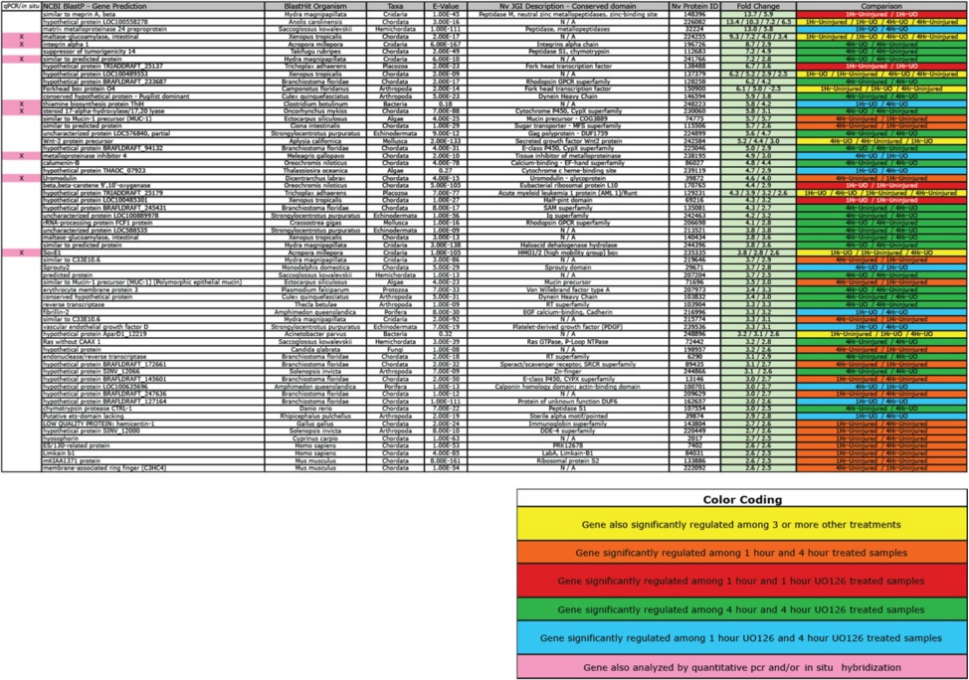
Comparison of genes identified through microarray analysis that are upregulated by injury and/or down-regulated by U0126.

To determine the transcriptional input necessary for stimulating a wound healing response, we used a *Nematostella* genome-wide microarray (Nimblegen, Inc., Iceland) to identify target genes involved in wound healing. We isolated mRNA from uninjured polyps, as well as from injured animals at one hour (early response genes) and four hours (late response genes). To determine how ERK signaling effects mRNA transcription during wound healing, we also collected U0126-treated animal mRNA one and four hours after injury for comparison. Genes organized in Figure 4 were identified in our microarray analysis and found to exhibit higher levels of expression due to injury activation and/or drug treatment. These genes are a subset of genes from Additional file 8, and represent genes that were found among multiple comparisons (temporally and/or drug-treated). A complete set of genes with every fold-change comparison can be found in Additional file 8 (RawBlast2GoData), along with analyzed Blast2Go results (Gene Ontology prediction and top BLAST hit). Genes in Figure 4 are organized in relation to the highest fold-change expression, and each gene is identifiable by its protein identification number from the JGI *Nematostella* genome website [32]. 

The genes with the highest up-regulation after injury and during wound healing were dominated by peptidase activity (148396, 32224, 112683, 107554) or moderators of MAPK signaling including alpha-integrin (196726) and g-protein signaling (128258, 206698, 72442). The primary transcription factors activated by injury were Fox O-related (138488, 150900), *Sox E1* (235335) and *Runt* (129231). A number of growth factor-related genes were also activated, including Wnt2 (242584), platelet-derived growth factor (239536) and sprouty (29671). Interestingly a number of genes related to mucus proteins or mucosa tissue were also highly up-regulated, including maltase-glucoamylase (224255) and mucin-1/2 precursors (74775; three others in the ‘Complete Dataset’ in Additional file 8). This cohort of genes occupies the full range of MapK signaling components from the immediate sub-nuclear activation (through phosphorylation) of MAPK-specific transcription factors to the extracellular activity of peptidases and across the membrane-bound regulators like G-proteins and integrin.

### Quantitative PCR reaffirms microarray findings

**Figure 5:**
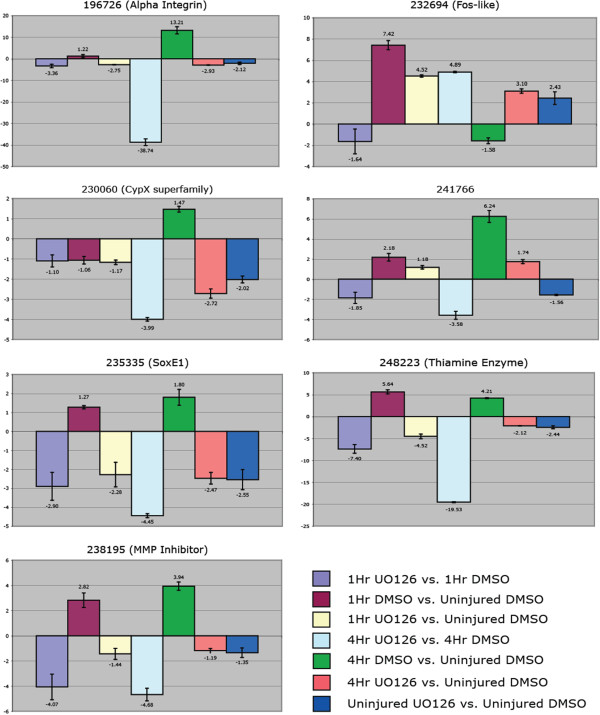
**Relative fold-change quantification of mRNA transcript levels confirms targets identified by microarray analysis.** Quantitative PCR of seven representative genes showing a similar affinity to U0126 as identified by microarray analysis. Genes were standardized against the house-keeping gene NvGADPH and confirmed with a second gene, NvRiboPro. Values <1 or > -1 are insignificant.

A total number of 17 genes were analyzed by quantitative PCR (qPCR) and/or by *in situ* hybridization to confirm microarray results. Genes were selected for qPCR based on their genomic sequence size and if the desired PCR primer conditions could be met for qPCR. The seven genes shown in Figure 5 represent genes that exhibited high fold-change levels from the array due to U0126 treatment and/or temporal variation. The Fos-like gene (232694) exhibited increased expression at one hour after injury and appeared to be up-regulated at four hours by U0126, where all other genes in Figure 5 were greatly reduced in relation to U0126. Seven additional genes (138488, 86916, 140525, 238642, 39805, 37059 and 98391) were characterized by qPCR (data not shown) and followed similar up- or down-regulation patterns as described by microarray analysis. Only a single gene (170407) identified as a potential candidate by microarray analysis exhibited little change by qPCR. This gene was only identified in our comparison between injured animals at four hours versus uninjured animals and had a relatively low fold change (2.9×).

### *In situ* hybridization of target genes confirms microarray results and localize around the site of injury

**Figure 6:**
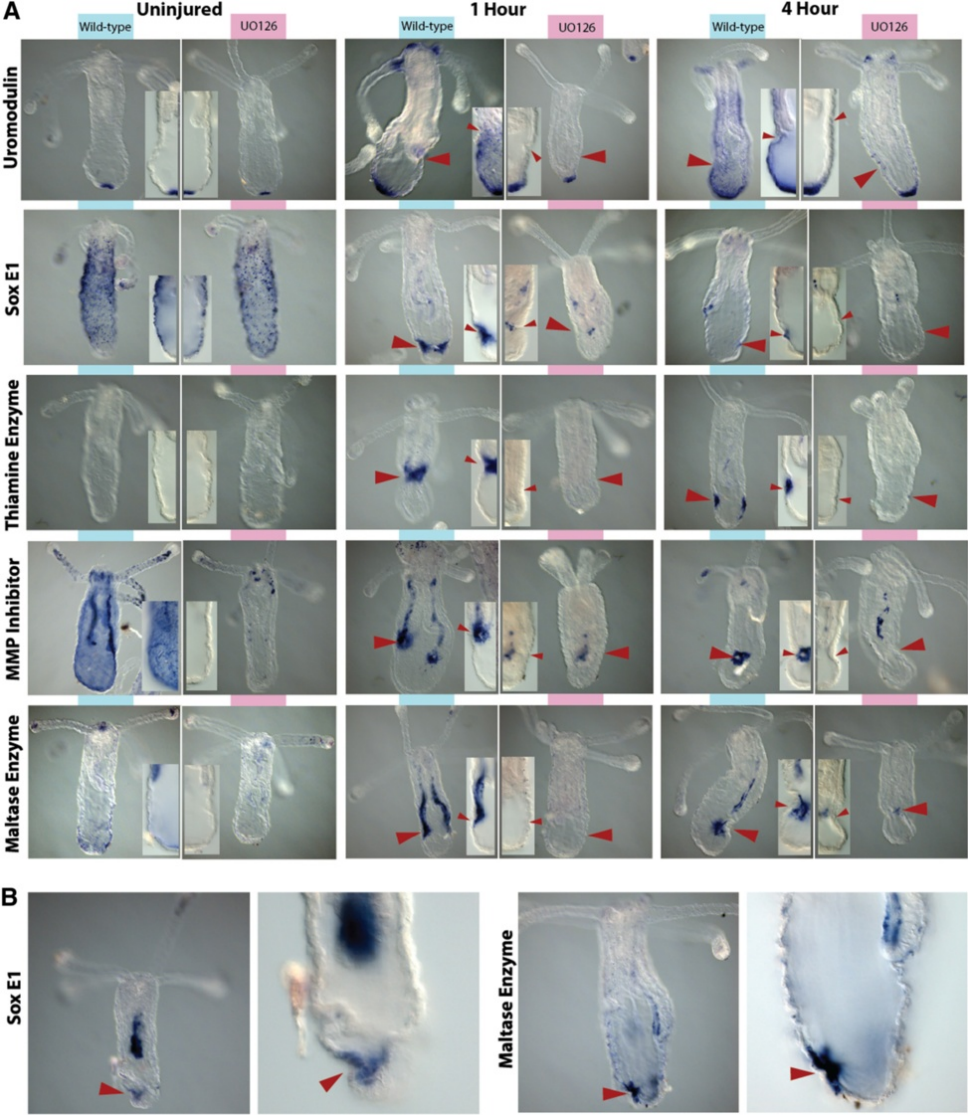
**Multiple signals are derived from different tissues during wound healing.** A comparison of different genes identified by microarray analysis and their expression profiles over time and in relation to U0126-treated samples. **(A)** Uromodulin (Row 1) is always expressed at the aboral pole and expands expression as a result of injury. Drug treatment appears to block the expansion of expression over time. SoxE1 (Row 2) is expressed in the endoderm around the injury site, appears down-regulated by U0126 and is expressed broadly in the endoderm, and in a potentially salt-and-pepper pattern in cells within the ectoderm of wild-type uninjured individuals. The thiamine enzyme (Row 3) is expressed primarily in the ectoderm of only injured animals. The matrix metalloproteinase (MMP) inhibitor (Row 4) is expressed within the mesenteries as well as the endoderm surrounding the site of injury. Wild-type expression is found throughout the endoderm and is reduced in all U0126-treated samples. The maltase enzyme (Row 5) is also restricted to the mesenteries and endoderm of injured animals. Wild-type expression is lowly expressed in the endoderm, mesenteries and tentacle tips. **(B)** Removal of U0126 reinitiates gene expression at the site of injury. U0126 was removed after four hours, then animals were allowed to heal for another four hours before fixation. Expression of SoxE1 and Maltase enzyme look strikingly similar to that in animals at four hours in **A**. All drug-treated samples showed reduced expression. Inset pictures compare control versus drug-treated samples. (Red arrowhead indicates the site of injury).

Uromodulin (39872), also known as Tamm-Horsfall glycoprotein, was normally expressed at the most distal portion of the aboral pole of uninjured animals (Figure 6, row 1, left). At one hour after injury, uromodulin was highly expressed around the wound ectoderm and expression had continued to expand in four hour animals (Figure 6, row 1, middle, right). The wild-type localized expression of this gene is not reduced following treatment with U0126 (Figure 6, row 1 left), U0126 appears to block the activation of *uromodulin* around the wound site, without reducing the endogenous expression around the aboral pole (Figure 6, row 1, middle, right).

SoxE1 (235335) belongs to the homeobox class of transcription factors and is normally expressed along the aboral ectodermal walls of the body column (Figure 6, row 2, left). Incubation with U0126 caused a slight reduction of expression in uninjured animals (Figure 6, row 2, left). Wounding caused broad ectodermal patterning to become reduced, and local expression near the injury site occurred in animals examined at both one and four hours after puncture (Figure 6, row 2, middle, right). Exposure to U0126 during wound healing thus reduced local expression (Figure 6, row 2, middle, right).

The thiamine biosynthesis enzyme (248223) was only expressed after injury and was localized to the ectodermal layer cells immediately around the site of injury (Figure 6, row 3). This gene also appeared to be controlled by ERK signaling because expression during injury was down-regulated in U0126 animals (Figure 6, row 3, middle, right). We did not detect any endogenous expression in uninjured animals (Figure 6, row 3, left).

The inhibitor of matrix metalloproteinase gene (238195) was broadly expressed throughout the endoderm and drastically reduced in U0126-uninjured animals (Figure 6, row 4, left). Expression was localized in the body column and tentacle endoderm as well as the mesenteries. In injured animals at both one and four hours, expression was localized to a circle within the endoderm around the injury site and was also up-regulated in the mesenteries (Figure 6, row 4, middle, right). U0126 reduced local expression around the injury site, but some expression remained within the mesenteries.

The gene encoding a maltase-like enzyme (224255) was lightly expressed compared to within injured animals, and localized to the aboral endoderm, tentacle tip endoderm and near the mouth (Figure 6, row 5). Puncture injury to the aboral region induced expression throughout the mesenteries and locally at the site of injury (Figure 6, row 5, middle, right). Expression in the mesenteries appeared to be greatest at one hour after puncture, whereas expression around the injury was higher at four hours after injury. U0126 treatment appeared to dissipate staining in both the mesenteries and locally at the site of injury.

### Animals recover gene expression and wound healing upon drug removal

We determined that removal of U0126 allowed injured animals to begin healing (Figure 3F), therefore we wanted to know if drug removal also restores normal expression of wound healing targets. We exposed punctured animals to U0126 for four hours after injury, then washed and incubated for another four hours without drug in 1/3× seawater. Removal of U0126 reinitiated the normal expression of target genes (Figure 6B). Both SoxE1 (235335) and the maltase enzyme gene (224255) exhibited expression domains comparable with wild-type punctured animals at four hours after injury. SoxE1 expression was greater within the endoderm in these washout experiments, but normal ectodermal staining around the wound was exhibited. The maltase enzyme was also expressed around the site of injury and mildly expressed within the mesenteries, comparable to wild-type animals. These experiments demonstrate the level of morphological and transcriptional recoverability after drug removal, and suggest that these target genes are necessary for wound healing.

### Microarray targets were also activated prior to oral and aboral regeneration

**Figure 7:**
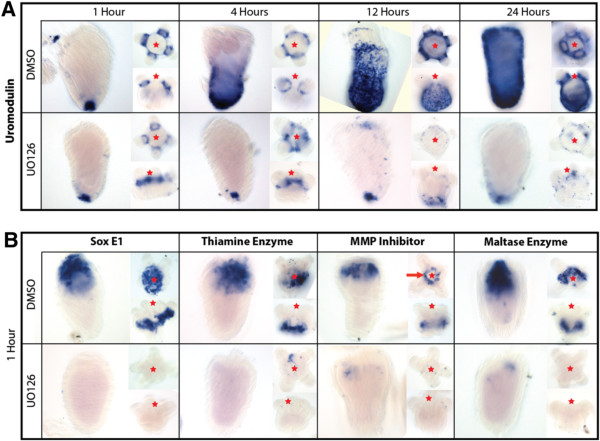
**Similar activation of wound healing transcripts during head regeneration.** All five genes identified by puncture assays were also up-regulated during wound healing, prior to regeneration. **(A)** Time series of expression levels of uromodulin transcripts during oral and aboral wound healing events. One hour after injury, uromodulin localizes to the aboral pole of tissue that is regenerating oral structures, and around the base of the tentacles in tissue regenerating aboral structures. U0126 blocks the normal activation of uromodulin during regeneration. Uromodulin appears to be highly expressed in the aboral ectoderm during both oral and aboral regeneration. **(B)** All four genes exhibit the same expression domains as previously identified during puncture analysis. SoxE1 and the thiamine enzyme are both expressed along the ectoderm at the injury site in both oral and aboral regenerates. Similar to puncture assays, the matrix metalloproteinase (MMP) inhibitor is highly expressed in the endoderm at the site of injury in a circle pattern. The maltase enzyme also localizes to the endoderm in both oral and aboral regenerates. Incubation of tissue in U0126 appears to reduce expression in all samples. All time points contain a lateral view of the aboral regenerate, an oral-aboral view of the oral regenerate, and a lateral view of an oral regenerate. (Red star indicates the position of the oral side of the animal, red line indicates circular expression).

Our findings through microarray analysis, qPCR and *in situ* hybridization suggest that we identified several genes utilized during aboral wound closure. To determine if our identified targets also play a role in wound healing before regeneration, we conducted *in situ* hybridization of animals that underwent bisection along the oral-aboral axis in comparison to animals impaired by U0126 (Figure 7). Similar to results obtained by our puncture assay, the uromodulin-like gene was expressed in the aboral domain immediately after injury and expression appeared expanded at four hours after injury (Figure 7A). This appears to be slightly delayed from the timing of puncture experiments. In oral halves, expression was found immediately around the base of the tentacles, similar to puncture experiments. Over time, expression in both the regenerating oral and aboral halves expanded from a localized expression (aboral pole or base of the tentacles) to a broad expression encompassing most of the ectoderm except the tentacles. Treatment with U0126 resulted in maintained endogenous expression of the gene along the aboral pole or base of the tentacles and, similarly to puncture assays, limited the expansion of gene expression associated with injury. Expression of uromodulin was maintained through the first 24 hours when proliferation is known to begin [16].

The four other genes, *SoxE1*, thiamine enzyme, matrix metalloproteinase (MMP) inhibitor and the maltase enzyme, all exhibited expression immediately after injury in both oral and aboral halves (Figure 7B). *SoxE1* was expressed along the ectoderm at the site of injury in both oral and aboral regenerates. The gene encoding for thiamine enzyme also localized to the ectoderm immediately surrounding the site of injury. The MMP inhibitor and maltase enzyme were expressed in the endoderm of regenerates. The MMP inhibitor exhibited a ring-like expression around the wound (Figure 7B, red arrow) similar to puncture experiments (Figure 6A). The maltase enzyme was highly expressed within the endoderm at the site of injury. Each of the four genes were analyzed at four, 12 and 24 hours but did not exhibit drastic changes in localization domain, although they exhibited lower expression at 24 hours (data not shown). Samples that were incubated with U0126 had decreased or absent expression.

## Discussion

Comparative embryological studies using *Nematostella vectensis* have shown that many molecule components utilized during early embryogenesis of deuterostomes were present in the cnidarian-bilaterian ancestor and remain today [9,33,34]. This suggests that the signals patterning during early embryonic development have an ancient origin and exhibit a strong level of conservation over evolutionary time. The healing of the wound in the epithelial layer after injury is a necessary process; disruption of this process is known to inhibit regeneration [2-5]. Therefore, to gain a better understanding of the regenerative potential between species we must first understand what signals activate wound healing and allow for the step-wise activation of regeneration. Characterization of the wound response in a diverse set of animals will provide clues into why so many animal lineages have lost the ability to regenerate as a result of defective wound repair.

In *Nematostella*, an injury is capable of initiating regeneration [16]. In the current study, the wound healing response in *Nematostella* was characterized to determine the cellular and molecular components necessary for activating a regenerative response. Our findings suggest that the wound healing response consists of an early cellular response and a late growth response that leads into the proliferative response necessary for regeneration.

### The early cellular response during wound healing

#### Peptidase activity and their inhibitors, regulators of cellular dynamics during wound healing

Growth factors play an instrumental role in the activation of wound healing [35]. The first signals present after injury in vertebrates are growth factors distributed to the site of injury through the release of blood and platelets. In *Nematostella*, we see an activation of similar growth factors (fibroblast, epidermal and vascular endothelial/platelet derived), but this activation appears hours after the initial injury. We have identified a number of early up-regulated MMPs that may act in releasing these growth factors to initiate synthesis. MMPs are a subset of peptidase enzymes that utilize calcium or zinc ions for activation and are known for their ability to release adhesion complexes between cells. Together with tissue inhibitors of metalloproteinases (TIMPs), which block MMP activity, cells are able to coordinate cell movement through regulation of adhesion molecules [36]. MMPs can also function to release growth factors or modify growth factor receptors during wound healing, where TIMPs are also thought to block pathogen MMP activity to prevent infection [37,38]. Similar to this study, MMPs have been identified in wound healing studies on *Hydra*[39], planarians [40], axolotl [41], mouse [42] and human skin [43]. Our findings suggest that numerous metalloproteinases are activated upon injury, while the Nematostella gene encoding for tissue inhibitor of metalloproteinase *NvTIMP* is expressed locally around the injury site (Figures 6 and 7). *NvTIMP* may suppress MMP activity around the wound to solidify the damaged tissue, creating a gradient of mechanical stiffness. A localized rigid cellular environment can act as a signal to promote cellular migration, otherwise called durotaxis, or the migration of cells towards an area of greater rigidity [44,45]. Although *NvTIMP* could be used for pathogen-host prevention, it may serve as a signal for cellular migration in *Nematostella*. Our microarray results suggest that metalloproteinase activity is a major component to wound healing in many different animals with various levels of regenerative capacity.

The primary events taking place during early wound healing of *Nematostella* appear to be cellular shape change, migration, adhesion and death. After any injury, a number of stimuli (for example, stress, mechanical forces, bacterial invasion, fluid loss) become factors in the regulation of the wound healing process. ERK signaling is known to play an important role in the regulation of apoptosis and bud formation in *Hydra*[46-48], and is necessary to establish blastema formation during head regeneration in planarians [49]. We have established that ERK signaling is activated immediately after injury in *Nematostella* and is necessary for proper wound healing. Although ERK signaling acts as an early response element among these three taxa, several novel genetic components to the *Nematostella* wound healing program may provide a significant advantage compared to other systems that lack a high regenerative capacity, other than simply having highly proliferative I-cells (*Hydra*) or neoblast cells (planarians).

#### Apoptosis is active throughout all phases of *Nematostella* wound healing

**Figure 8:**
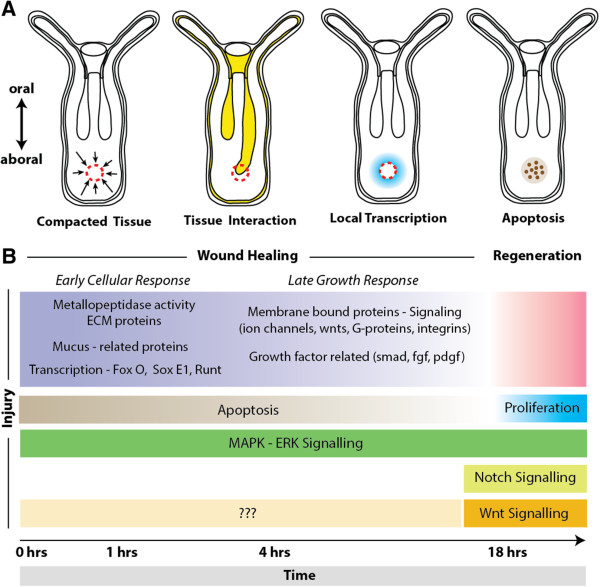
**Summary of the signals, pathways and cellular behavior of *Nematostella *wound healing. (A)** Potential tissue interactions that initiate the transition from wound healing to regeneration. Puncture wound healing involves collapsing of tissue due to the compromised tissue walls, resulting in cells becoming compacted in local proximity to the wound. Tissue like the mesenteries could provide local signals to aid in initiating wound healing as it comes in contact with the injury site in both puncture assays and decapitated animals. Wound healing-related gene expression is found locally around the site of injury and along the mesenteries during healing. Apoptosis is a known regulator of future proliferation during regeneration and is active during *Nematostella* wound healing. **(B)** Timeline of events leading to a regenerative response. Wound healing and regeneration in *Nematostella* appear to be developmentally separable processes, where wound healing initiates regeneration. Apoptosis is activated immediately after injury, where proliferation may be the key transition for a regenerative response. MAPK-ERK signaling spans over the whole regenerative process, potentially regulating cellular movement and/or recognition during wound healing and proliferation during regeneration. We did not find any evidence that Notch signaling effects the wound healing process, but rather inhibited head regeneration. The Wnt planar cell polarity pathway is likely to effect wound healing and is known to regulate axis formation during regeneration.

Apoptosis is the known driver of future proliferation in a number of species after an injury occurs [30]. Cell death appears active throughout the early and late injury response in *Nematostella* (Figure 2E, Figure 8B). *Hydra* studies show that inhibition of apoptosis is known to inhibit regeneration without wound healing defects, and ectopic activation of apoptotic signals can induce a second head to grow [29,48]. Contrary to the findings of Chera *et al*. [48], we did not see a decrease in apoptotic signal due to inhibition of ERK signaling. Interestingly, blocking notch signaling in *Hydra* does not inhibit head regeneration [50], where inhibition during embryogenesis of *Nematostella* causes tentacle deformities [51] and loss of tentacles during regeneration (this study). This suggests that although there is a conserved cell behavior necessary for regeneration, the modes of regeneration may be quite different even within the same phylum. Furthermore, activation of apoptotic signals during wound healing could be necessary for initiating proliferation, but inhibition of normal wound healing by U0126 did not result in a loss of apoptosis, suggesting other signals along with apoptosis initiate a regenerative response.

#### Glycoproteins play an important role in *Nematostella* wound healing

Immediately after injury, animals exhibit a ‘deflated collapsed state’ due to failure to maintain positive pressure in the gastrovascular cavity [18]. This collapsed state may help push damaged areas together, allowing for rapid closure of the wound (Figure 8A). During these early hours after injury, the endodermally derived mesentery structures often acted to plug the wound (Figure 2B; Additional files 2 and 3; Figure 8A). A sticky mucus-like substance was excreted from the wound (potentially from mesentery interactions) and may aid in closing the wound (Figure 2B; Additional file 4). A puncture injury inhibits peristaltic movements down the oral-aboral axis immediately around the site of injury (Additional file 1). Peristaltic movement likely provides nutrients and oxygen exchange throughout the body and inhibition leaves animals in a compromised state. Therefore, the interaction of mesenteries and/or mucus could help animals temporally stop water from leaking from the gastrovascular cavity, and may act as a cue to initiate local wound healing around damaged tissue.

A number of mucin-related glycoprotein genes are activated early during *Nematostella* wound healing (Figure 4). Mucins are classified into two groups, transmembrane or secreted gel-forming mucins; phylogenetic analysis suggests *Nematostella* has primarily gel-forming mucins [52]. Gel-forming mucins are thought to provide a protective layer for underlying mucosal epithelial cells and are known to be expressed after damage to mucosal tissue. Mucin (muc-2)-deficient mice show impaired gastric healing [53,54]. Future experiments may reveal an interesting link between mucosal tissue and the role of mucins in regenerative animals, and the possibility of discovering other genes capable of accelerating wound healing [55].

We identified one uromodulin-like glycoprotein in *Nematostella*, an ortholog that is also highly up-regulated in the regenerating epithelial layer of axolotl limb regenerates [41]. Expression of this gene was restricted to the wound ectoderm in *Nematostella*, and appeared to spread laterally away from the wound over time. Interestingly, wild-type expression of this gene was localized to the aboral portion of the animal (Figure 6A), the position of the apical tuft during larval development. The apical tuft region of the animal is often associated with habitat selection and potentially bacterial recognition in corals during metamorphosis [56]. In mammalian systems, uromodulin protein is found in urine and is locally synthesized in epithelial cells of the kidney. Uromodulin-knockout mice have a great susceptibility to bacterial infection and this gene is thought to regulate inflammatory signals to allow healing [57,58]. Although these data are all based on studies of the kidney, this gene may be a part of a conserved pathway of wound healing that operates for regenerative and non-regenerative species.

#### Signals from the endoderm direct wound healing events

A number of conditions suggest that the endoderm in cnidarians may act as the driving force for wound healing. During wound healing of *Hydra*, the endoderm closes prior to the ectodermal layer [59]. In *Nematostella*, two genes analyzed by *in situ* hybridization (for MMP inhibitor and maltase enzyme) exhibited mesentery staining during wound healing (Figure 6A), and were localized to the endoderm during regeneration (Figure 7B), suggesting signals generated here may help guide wound healing. Sox genes have been found in all animals and SoxE-related genes are localized in developing endoderm during gastrulation in *Nematostella* and *Acropora millepora*[60,61]. A single SoxE-like gene is also expressed during early wound healing events, suggesting that some of the same genes utilized during gastrulation movements could be reutilized during healing. SoxE expression shows a conical shape of tissue within the endoderm, suggesting these cells are contracting towards the injury site (Figure 6A). Sox9 genes (group E members) have been implicated in neural crest development, a process often compared to gastrulation and wound healing [62,63]. Sox9 is also a stem cell marker in epidermal cells and is activated during healing and regeneration in mice [64].

We found that cells around the wound exhibited increased levels of actin at two hours after injury (Figure 2B). It appeared that the actin cytoskeleton was recruited to close the wound through filopodial extensions into the wound margin, rather than the actin purse-string model described in *Drosophila*[22]. In a number of samples, actin filopodia could be seen connecting the two injury sites (Figure 2C). It has been noted that actin filopodia extend into the blastocoel during *Nematostella* gastrulation [65] and both wound healing and gastrulation are halted by inhibition of ERK signaling. Overall, several lines of evidence suggest that wound healing could share many cellular and molecular behaviors and genes also used during gastrulation.

#### A bacterial-derived thiamine synthesis enzyme is highly expressed post-injury

We identified a cnidarian-specific gene (thiamine synthesis enzyme), an enzyme utilized during the synthesis of vitamin B, a deficiency of which has been linked to wound healing defects [66]. This gene is found on scaffold 466 in the *Nematostella* genome, was represented in expressed sequence tag resources utilized in genome construction [8], and was confirmed to be highly up-regulated after puncture injury by qPCR and *in situ* hybridization (Figures 4, 5 and 6). However, thiamine enzymes are not known in animals, and BLAST analysis only finds similar representatives of this gene in bacteria and *Acropora digitifera* (Additional file 9). This gene appeared to only be activated after injury, and BLAST analysis did not reveal other components of vitamin B synthesis (data not shown). Together, these data suggest that this gene likely incorporated into the *Nematostella* genome through lateral gene transfer and may aid in production of vitamin B by symbiotic bacteria within the animal, or alternatively have a novel wound healing function independent of vitamin B. Functional analysis of this gene may reveal an interesting novelty that has provided an evolutionary advantage for cnidarian healing and regeneration.

### Late wound healing response

#### A single α-integrin is up-regulated in response to wound healing

At four hours after injury, we began to see components of cell membrane signaling (integrins, G-proteins and a wnt ligand) as well as activation of growth factors*.* Integrins are mediators of focal adhesion complexes and numerous α- and β-subunits are activated or up-regulated during vertebrate wound healing [67]. *Nematostella* and closely related coral species have fewer numbers of integrins than vertebrates [68]. We identified a single α-integrin up-regulated at four hours after injury, with significant expression change in relation to the mitogen-activated protein kinase kinase (MEK) inhibitor, U0126. Although we do not know the location of expression, we predict that it will be found in a similar location to *SoxE* because these two genes are known to be in overlapping regions of the presumptive endoderm during gastrulation in *Acropora millepora*[61,68]. Due to functional redundancy and lethality of integrin knockouts in vertebrates [67], *Nematostella* could be a valuable system to discern the functional relationship of integrins and wound healing.

The activation of integrin is closely associated with regulation of calcium and ionic balance [69] and a number of calcium-related proteins (86027, 216996, 100701) were activated at four hours in *Nematostella*. In *Hydra* and axolotl, the EF-hand calcium-binding are associated with the wound response [40,41] and are thought to function during cnidarian morphogenesis [70]. EF-hand motif-containing genes regulate filopodia formation in migratory cells [71], and are a cellular component known to participate in *Nematostella* gastrulation [65]. Although we have made several comparisons between the potential parallels of gastrulation and wound healing, a direct comparison of the transcriptional repertoire of genes utilized in both gastrulation and wound healing (in relation to U0126 treatment) would need to be done to confirm our findings.

#### Expression of growth factors is delayed in the *Nematostella* wound healing process

Based on the timeline of gene activation (Figure 8B), our results suggest that MMPs may have a conserved role in cell movement and may act in releasing growth factors to initiate synthesis. The growth factor *PDGF* (239536) has not been characterized in detail in *Nematostella*, but other fibroblast growth factor-related genes are thought to regulate axial patterning and cell specification [72-74]. Interesting, a single *TGF-β* gene was identified to be down-regulated in wild-type animals at one hour compared with U0126-treated individuals. Tight regulation of *TGF-β* and downstream targets is suggested as a possible means to resolve excess scar formation [31]. Data found in this paper suggest that there is a delay in growth factor initiation, unlike many vertebrate systems in which growth factors arrive immediately after injury through the circulatory system [35]. Comparing how growth factors are activated in different model systems may be crucial for understanding how wound healing varies in animals with different regenerative capacities. Based on the timing and activation of proliferation during regeneration, it is likely that this later stage of wound healing prepares the tissue for proliferation (Figure 8B). Future studies will be necessary to determine which genes are functionally responsible for the initiation of regeneration.

## Conclusions

Wound healing and regeneration are separable developmental processes. Here we suggest that healing from a puncture wound takes roughly six hours after injury to allow for injured tissue to become functional again. Some time between 12 and 18 hours, signals from healed tissue activate a program initiating proliferation and regeneration [16]. We have shown that the same genes that heal puncture wounds also are activated after oral-aboral bisection. Apoptosis is activated immediately after injury and could provide signals for head regeneration, although ectopic activation of apoptotic signals in the aboral zone does not induce a second axis. Interestingly, notch signaling only effects head regeneration whereas inhibition of ERK signaling is capable of blocking healing and regeneration (Figure 8B). We propose that in *Nematostella* the key transition from wound healing to a state of regeneration is the activation of cell proliferation. ERK signaling is necessary for the initiation of the early wound healing response in *Nematostella* and is closely linked to the activation of proliferative cells in other highly regenerative invertebrate model systems [46,47,49,75], After the first wave of ERK-generated signals, later stages appear to prepare the tissue for proliferation of lost structures. This study uncovered a potentially important role of glycoproteins (mucins) during wound healing and found a novel anthozoan-specific thiamine biosynthesis enzyme utilized during healing. We have demonstrated that whole genomic microarray analysis is a powerful way to identify new targets of developmental processes. Our study is the first to identify and characterize genes involved during wound healing in *Nematostella* and is relatable to multiple model systems of regenerative biology.

## Materials and methods

### Animal care, cutting and puncture assays

*Nematostella* polyps raised in 1/3× seawater [76] were collected at day 14 after fertilization. Animals underwent two feedings of *Artemia* between day seven and day fourteen after fertilization, and then were starved for seven days to minimize non-specific staining due to food particles in the body cavity. Juvenile polyps were used due to their small size and ease of visualization and because they can regenerate lost structures similar to adults. Animals were cut in 2 mm thick silicon-coated dishes (SYLGARD–184, Dow Corning, Inc.). The silicon-coated dish was used to create a cutting/poking surface that did not damage the tools used. Glass needles for puncture assays were formed from capillaries (World Precision Instruments – TW100F4) using a needle puller (Model P97, Sutter Instrument Co.). All transverse cuts were done with a cornea scalpel and were performed near the base of the pharynx. Cutting experiments were done in all regeneration experiments; puncture experiments were used to assay components of wound healing. A standardized protocol for injuring animals was developed, similar to puncture assays in *Drosophila*[22], to allow for easy visualization of cellular events during wound healing. Punctures were centered between the base of the mesenteries and aboral tip of the animal (Figure 1B). We chose this area to assay wound healing targets because of the thin tissue layer that can be easily visualized microscopically. This area is also highly regenerative in *Nematostella*[13], being the approximate location of bud formation, and serves as a comparative point with other cnidarians such as *Hydra*.

Animals used in drug treatment experiments were soaked for one hour prior to injury. The drugs U0126 (Cat. #U120; Sigma, Inc.) and DAPT (Cat. #D5942; Sigma, Inc.) were both used at 10 μM concentration dissolved in 1% dimethyl-sulfoxide (DMSO). These two drugs have been shown to knockdown components of MapK (U0126) and notch (DAPT) signaling in *Nematostella*[72,51]. For experiments lasting longer than 12 hours, drugs were replaced every 12 hours until the termination of the experiment. All experiments were conducted at 25°C in the dark to prevent degradation of the inhibitors. For recovery experiments, animals were washed three times with 1/3× seawater, and then placed back in the dark at 25°C.

### Techniques utilized in wound healing assay

#### TUNEL assay for apoptosis

We used the DeadEnd Colorimetric TUNEL kit (Cat. #G7130; Promega, Inc.) to determine the distribution of apoptotic cells over time. Although the manufacturer’s protocol was designed for tissue sections, we performed the following for whole mount preparations: animals were relaxed by gently adding 7% MgCl_2_ to the 1/3× seawater; animals were fixed with 4% paraformaldehyde in 1/3× seawater for one hour at room temperature; animal tissue was washed five times with PBS with 0.2% Triton-X100 (PBT); fixed polyps were permeablized with proteinase K for 20 minutes as specified in the protocol; tissue was then washed twice with PBS and re-fixed with 4% paraformaldehyde in 1/3× seawater for one hour at room temperature; the tissue was washed five times in PBS; manufacturer’s protocol was followed for equilibration, biotinylation and SSC washes of the tissue; we added two extra 0.3% hydrogen peroxide washes (three times total for 15 minutes each) to help quench endogenous peroxidase activity; tissue was then washed twice in PBS; streptavidin horseradish peroxidase antibody was incubated overnight at 4°C; due to the larger amount of tissue used (rather than thin tissue sections), a larger volume of developing solution was needed, therefore signal was developed using DAB (Cat. #11718096001; Roche, Inc.) rather than kit components. The developing reaction was stopped by washing with PBS. Samples were then cleared in an 80% glycerol solution containing Hoechst (Cat. #H1399; Life Technologies) to label nuclei. Samples were quantified by measuring a 50 × 50 μm area centered around the site of injury (aboral region in controls) and the number of nuclei in this region was compared to the number of DAB positive cells (Additional file 6).

#### Mucus staining

We attempted to stain *Nematostella* mucus with the histological stains Alcian blue (acidic mucins) and Periodic acid-Schiff reagent (neutral mucins). These stains are used to visualize mucins present in mucosal tissue in vertebrates [77]. Samples were compared over time and against individuals that were exposed to U0126. Samples were gathered one, four and twelve hours after oral bisection and compared to uninjured animals to determine if there was an increase of mucus after injury and if the drug U0126 had any effect on mucus production. Samples were pre-incubated in gelatin-coated dishes with 1% DMSO (controls) or 10 μM U0126 (experimental) dissolved in 1/3× filtered sea water for one hour prior to injury. Juvenile polyps were cut along the oral-aboral axis (Figure 1C) and incubated at 25°C in the dark. Prior to fixation, samples were relaxed with 7% MgCl_2_ to the 1/3× seawater. Samples were fixed by washing polyps three times with ice-cold 100% methanol for one hour at 4°C. Samples were rehydrated immediately after fixation in 60% methanol in distilled water (once), then 30% methanol (once), and finally PBT solution for five washes. Samples were transferred to glass three-spot dishes for subsequent staining. At this time samples were soaked in Alcian blue solution (pH 2.5) for 10 minutes, then washed three times in distilled water. Samples were soaked in 1% Periodic acid solution for 10 minutes, then washed three times with distilled water. Schiff’s reagent was added to each sample and was stained for approximately 20 minutes. Samples were then washed 10 times with distilled water, then dehydrated through a methanol series: 50% (twice), 75% (once), 85% (once), 95% (once) and 100% (twice). Samples were further cleared (three times) in Murray clear (1:2 benzyl alcohol:benzyl benzoate).

#### Phallacidin, Hoechst and phosphorylated-ERK antibody

We found that after six hours puncture wounds were no longer visible by transmitted light and therefore we used confocal microscopy to determine the timeline of events leading up to the completion of the wound healing process (Figure 2). Samples were relaxed and fixed as in our apoptosis protocol (above). We used Biodypy FL phallacidin diluted 1:100 (Cat. #B607; Life Technologies) in PBT to visualize F-actin, especially along cell boundaries. Nuclei were visualized by incubation in Hoechst diluted 1:500 in PBT. Samples were incubated in a mixture of phallacidin and Hoechst overnight at 4°C. Samples were washed three times in PBT then cleared with 80% glycerol.

We utilized an antibody against phosphorylated-ERK, (Cat.#4377; Cell Signaling Technology) to identify if p-ERK was activated during wound healing and regulated by U0126 (Figure 3G,H). In these samples, animals were punctured in the aboral region and allowed to rest for one hour prior to fixation. To maintain the phosphorylated activity, all phosphate buffers were avoided, and instead animals were washed (five times) in Tris-buffered saline with 0.1% Tween20 (TBST buffer). Specimens were blocked in 5% normal goat serum in TBST buffer overnight at 4°C. Samples were incubated in p-ERK antibody at 1:200 overnight at 4°C. The antibody was removed and samples were quickly washed three times in TBST buffer, followed by three additional washes of 10 minutes each. Specimens and antibodies were again pre-blocked for one hour at 4°C. A secondary antibody, Alexa Fluor® 488 goat anti-rabbit (Cat.#A11008; Life Technologies), was used at 1:250 and placed in 5% normal goat serum in TBST buffer overnight at 4°C. The secondary antibody was removed and samples were washed three times in TBST, then cleared in 80% glycerol.

### Imaging

Many different techniques were attempted to immobilize living animals for visualization during wound healing, including increasing the viscosity of the media, creating small chambers for enclosure, and deciliation, but the best method was using negative pressure with one or two suction pipettes to hold animals in a fixed position. We used small capillaries (Cat. #TW100F4; World Precision Instruments, Inc.) attached to small transfer pipettes to gently create suction on the side of the polyp (see Additional files 1, 2 and 3). With this method, we were able to hold animals in a similar viewing plane for up to eight hours at a time. These animals were mounted under cover slips and sealed with Vaseline to prevent water evaporation during live imaging. Acridine orange was used as a counter stain in our experiments to help visualize structures that were not visible using transmission light microscopy. We prepared a 1 μM acridine orange solution (in 1/3× sea water) incubated animals for five minutes, and then washed the animals three times with 1/3× seawater before experimentation.

Time series images of regenerating head structures (Figure 1B) were taken on a Zeiss Axio Imager Z1 using a Hamamatsu (Orca-ER) camera with Volocity 5 software [78]. Photographs of drug-treated animals (Figure 3A-D) and apoptosis images (Figure 2E) were taken on an Axioscope 2 compound microscope using an AxioCam (HRc) camera with Axiovision software (Zeiss Inc., Jena, Germany). The mucus-labeling experiment (Additional file 5), time series of puncture wound healing (Figure 2A-D) and all additional videos were taken on a Zeiss 710 scanning laser confocal. Z-stacks images and time-series videos were compiled with Zen software (Zeiss Inc.). Images from the *in situ* hybridization experiments were captured using a Zeiss Axio Imager M2 using an AxioCam (HRc) camera and processed using Zen software. All figures were created using Adobe Illustrator (CS4).

### RNA and cDNA handling

A total of 300 polyps (for microarray) or 100 polyps (for qPCR) were used for one biological replicate for each assay. RNA extraction techniques and cDNA synthesis were the same as described in Layden *et al*. [79] and Röttinger *et al*. [80]. To maximize the abundance of wound healing transcripts, multiple puncture wounds were created in animals used for microarray and qPCR analysis. These animals received three puncture wounds along the oral-aboral axis. Therefore, these transcripts likely incorporate wound-related genes regardless of the body position. A single wound was formed in animals that were used for *in situ* hybridization and our other imaging studies.

### Microarray

Our 4-plex Nimblegen Inc. microarray chip consisted of 72,000 features, covering the complete *Nematostella* genome with three replicate oligonucleotide probes per gene. Samples were normalized and fold-change calculations were produced using Nimblegen Inc. software according to previous work [81,82]. All associated microarray files were uploaded to ArrayExpress [83] under the accession numbers A-MEXP-2380 (design file) and E-MTAB-2341 (protocol and data file). Treatments included uninjured animals (in 1% DMSO), animals one and four hours after puncture injury (in 1% DMSO), and animals after one and four hours in U0126 (in 1% DMSO) at a concentration of 10 μM. A total of two biological replicates per time point and treatment (DMSO versus U0126) were analyzed at 300 polyps sample. Due to the large number of polyps utilized and the laborious nature of each wound experiment, we chose to analyze only two biological replicates by microarray treatment and confirm these results by qPCR and *in situ* hybridization. In all drug treatment experiments, animals were pre-soaked for one hour prior to injury. A total number of 1,434 significant expression values exhibited a fold change of 2.5 or greater (Additional file 8). From this dataset we analyzed a total 830 protein sequences from the *Nematostella* genome via the Joint Genome Institute [32]. Each sequence was manually BLASTed against NCBI’s protein BLAST database [84] and we recorded the top BLAST hit, species, e-value and any predicted domains. To extract the maximum amount of data for each gene, we also gathered gene description information (column NvJGI Description - conserved domain, Figure 4) from the Joint Genome Institute website. All genes were also analyzed with Blast2Go [85] software, and added to our Additional file 8 (RawBlast2GoData).

### Quantitative PCR

qPCR samples were standardized with NvGADPH and NvRiboPro (Additional file 10). Primers for other genes were designed using MacVector [86] to amplify 75 to 150 base-pair fragments of the desired gene. These primers were then back-BLASTed against the *Nematostella* genome to make sure they only amplified a single region from the genome. We checked each primer’s efficiency with a dilution curve (10^-1^ to 10^-5^) to make sure their range was within the negligible value of 1.9 to 2.0. A total of three biological replicates consisting of 100 polyps per sample were analyzed. Relative fold-change values were calculated in Microsoft Excel and were standardized against our reference genes based on formulas from Livak and Schmittgen [87].

### *In situ* hybridization

All *in situ* hybridizations were based off the previous protocol for *Nematostella vectensis*[88]. Fixations were done in 1% gelatin-coated dishes to prevent tissue sticking to the plastic (sticking to plastic causes tissue damage and non-specific staining). Animals were fixed in ice-cold 4% paraformaldehyde with 0.2% glutaraldehyde in 1/3× seawater for two minutes, followed by 4% paraformaldehyde in 1/3× seawater for one hour at 4°C. DIG-labeled probes, ranging from 550 to 1,200 base pairs, were hybridized at 64°C for two days and developed with the enzymatic reaction of NBT/BCIP as substrate for the alkaline phosphatase-conjugated anti-DIG antibody (Cat.#11093274910; Roche, Inc.). Samples were developed for an equal amount of time and if no expression was visible a subset of samples remained in developing solution to determine if any expression was present.

## Abbreviations

DMSO: dimethyl-sulfoxide; MAPK: mitogen-activated protein kinase; MMP: matrix metalloproteinase; qPCR: quantitative polymerase chain reaction; PBS: phosphate-buffered saline; PBT: phosphate-buffered saline with .2% Triton-X100; TBST: Tris-buffered saline with 0.1% Tween20; TGFβ: transforming growth factor beta.

## Competing interests

The authors declare that they have no competing interests.

## Authors’ contributions

TQD and MQM were involved in project design. TQD conducted all experimental aspects of the project including microarray and qPCR analysis, in situ hybridization, staining protocols and confocal microscopy. TQD, NTK and MQM were involved in the synthesis of the manuscript. All authors read and approved the final manuscript.

## Supplementary Material

Additional file 1 **Time-lapse movie from 2 to 6 hours after injury.** Photos were taken every 15 seconds. Movies are played at four times the normal speed. This movie highlights the behavioral change in peristaltic movement during early aboral puncture wound healing. Over time, the wound closes and normal peristalsis can resume Click here for file

Additional file 2 **Time-lapse movie over the first hour after injury.** Photos were taken every 15 seconds. Movies are played at four times the normal speed. Animals were stained with acridine orange and fluoresced with 488 nm argon laser (with only 10% power). This movie shows the interaction between mesentery structures and the puncture wound immediately within the first hour of injury. Click here for file

Additional file 3 **Time-lapse movie over the first three hours after injury.** Photos were taken every 15 seconds. Movies are played at four times the normal speed. Animals were stained with acridine orange and fluoresced with 488 nm argon laser (with only 10% power). This movie shows the interaction of mesentery structures over the course of the first three hours after injury. Click here for file

Additional file 4 **Time-lapse movie over 15 minutes during the first hour after injury.** Photos were taken every 10 seconds. Movies are played at two times the normal speed. Animals were stained with acridine orange and fluoresced with 488 nm argon laser (with only 10% power). This movie shows a sticky mucus-like residue left from the injury site as the animal migrates out of the focal view. Click here for file

Additional file 5 **Mucin analysis during regeneration.** Animals were stained with Periodic acid and Schiff’s reagent which contains the fluorescent compound fuchsin. **(A)** Scanning laser confocal images of the head (oral region) show large round bundles of cells that are heavily stained with fuchsin (white arrow). **(B)** Structures similar to those found in the pharynx are found throughout the ectoderm in the aboral part of the animal (white arrow). **(C)** The brightest concentration of staining occurs at the base of the mesenteries; **(D)** little to no staining was found along the tentacles. **(E-E’)** Uninjured animals appear to have less fluorescent labeling found throughout the animal when individuals are exposed to U0126. **(F-H’)** Time series of mucus staining during wound healing after head removal. **(F-F’)** One hour after injury fluorescent staining appears greatest near the wound epithelium. **(G-G”)** A brighter amount of staining appears present at four hours after injury, while U0126 animals show wound healing defects and less staining. **(H-H’)** By 12 hours, little mucin staining is visible in controls, where U0126 animals still exhibit wound healing defects, but appear to have elevated mucin levels. Click here for file

Additional file 6 **Maintained apoptotic regulation during wound healing. (A)** Quantification of apoptosis and nuclei count of five different stages of juvenile polyps before and after injury. Samples exhibit relatively equal numbers of TUNEL-labeled cells, where uninjured animals express the lowest number. **(B)** Ratio of apoptosis compared to total nuclei count shows maintained apoptosis throughout early puncture events. Click here for file

Additional file 7 **Inhibition of notch signaling disrupts head regeneration.** Exposure of regenerating polyps to the notch inhibitor, DAPT, prevents head from developing new oral regions. Click here for file

Additional file 8 **This file contains all microarray data.** Each document is separated by a different tab. This is the excel file used to create Figure 4 in the paper. A KEY designates what the colors mean in Figure 4. Complete Dataset is the accumulated amount of genes that showed expression greater than 2.5-fold-change and were identified due to U0126 treatment or injury. Each subsequent tab (1Hr-Uninjured, 1 Hr-1HrUO, 4 Hr-Uninjured, 4 Hr-4 Hr UO) are individual comparisons of temporal and drug-interactions with injured and non-injured animals. The RawBlast2GoData tab is a full dataset of every comparison, including (1Hr-4Hr, 1Hr-4HrUO, 1HrUO-4Hr, 1Hr-4HrUO, 1HrUO-Uninjured, and 4HrUO-Uninjured). This dataset includes expression data from the array as well as Gene Ontology predictions and the top blast hit for each protein. The last two tabs are compiled from the “Complete Dataset” and contain a pie chart and the data used to make it. This pie chart demonstrates the relatedness of the wound healing response genes to their BLAST. Although it may be bias by the number on genomes in the NCBI database, it suggests that 40% of the genes are closely related to those of chordates. Interestingly 10% are related to bacteria, and 12% belong to other deuterostomes (hemichordates and echinoderms). From a blast perspective, over half the genes activated have a common potential relative in deuterostomes, although a more detailed phylogenetic analysis needs to be performed on a gene-by-gene basis. Click here for file

Additional file 9 **The thiamine enzyme from *Nematostella *is likely a cnidarian-specific gene, derived from bacteria.** This gene is found on scaffold 466 and spans positions 44637:52229. **(A)***Acropora digitifera* genes closely related to the thiamine enzyme identified in *Nematostella vectensis* and their associated homology. **(B)** Alignment of *Nematostella* and *Acropora* sequences. Click here for file

Additional file 10 Primer information for cloning. Click here for file
