# Supplementary figures and images for: Initiating a regenerative response; cellular and molecular features of wound healing in the cnidarian Nematostella vectensis
Source: BMC Biol. 2014 Mar 26;12:24. doi: 10.1186/1741-7007-12-24 (PMC4229989; doi:10.1186/1741-7007-12-24)

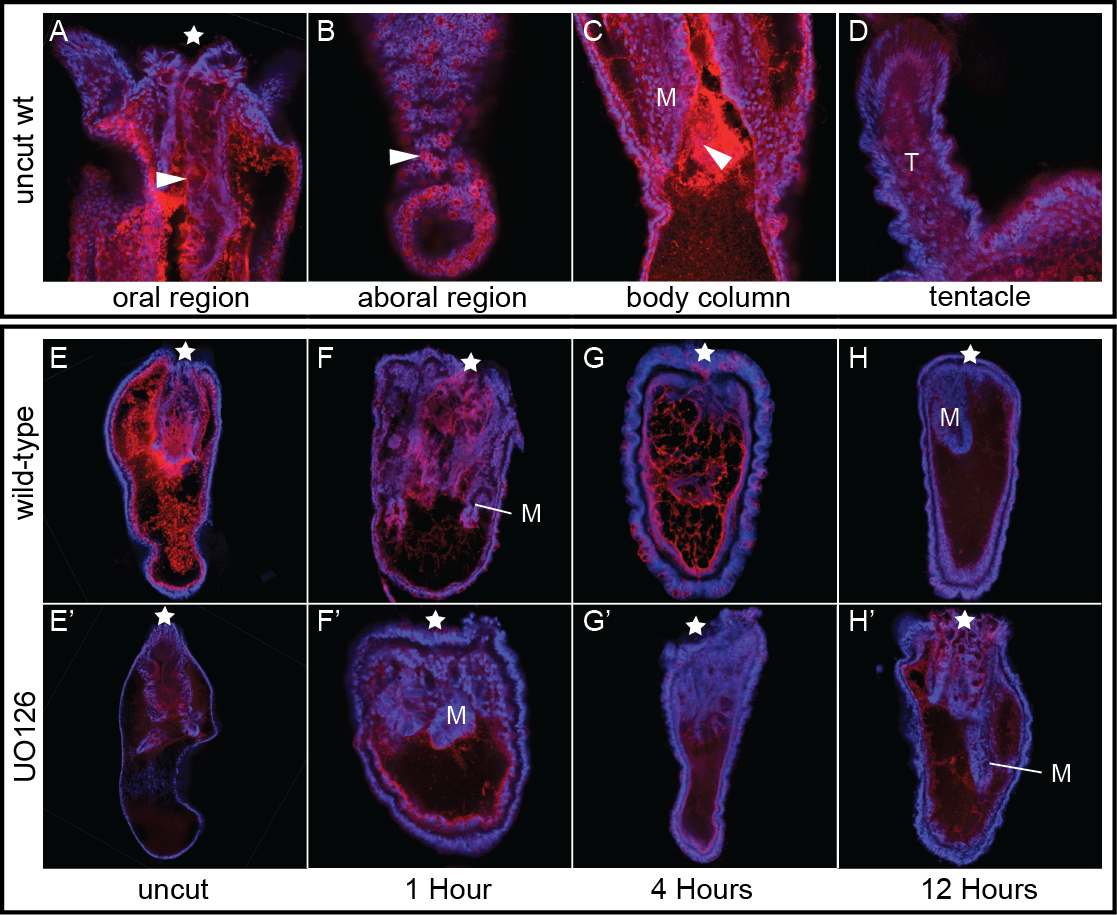

Supplement: Additional file 4 — Time-lapse movie over 15 minutes during the first hour after injury. Photos were taken every 10 seconds. Movies are played at two times the normal speed. Animals were stained with acridine orange and fluoresced with 488 nm argon laser (with only 10% power). This movie shows a sticky mucus-like residue left from the injury site as the animal migrates out of the focal view. [file 1741-7007-12-24-S4.png]

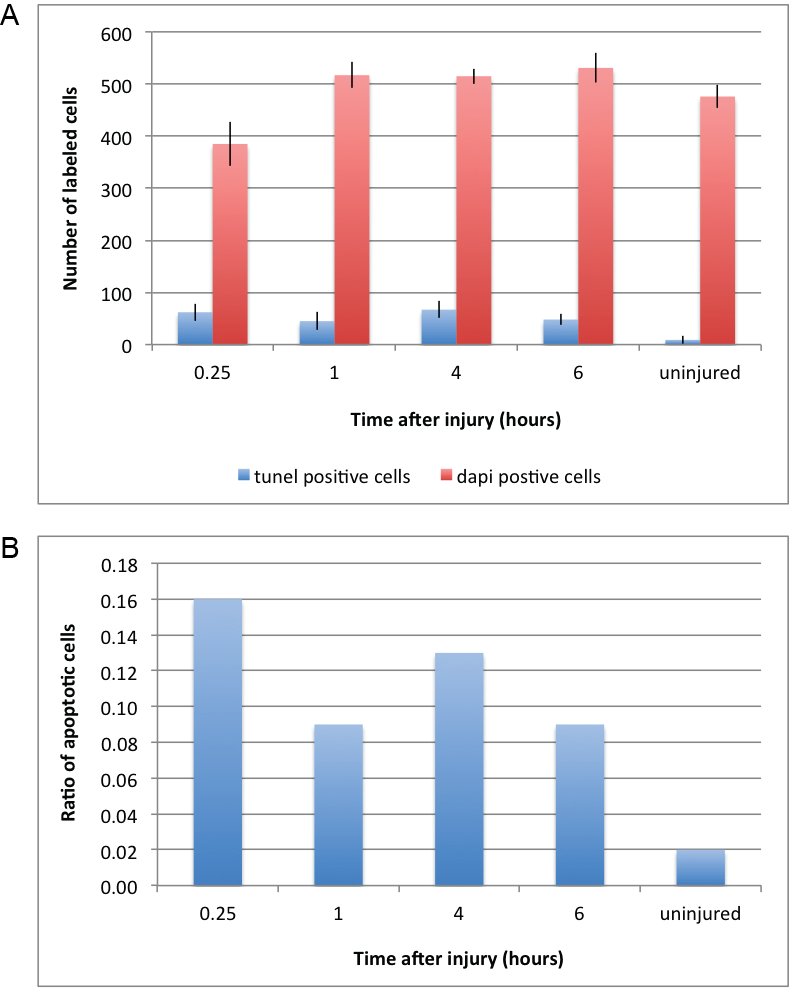

Supplement: Additional file 5 — Mucin analysis during regeneration. Animals were stained with Periodic acid and Schiff’s reagent which contains the fluorescent compound fuchsin. (A) Scanning laser confocal images of the head (oral region) show large round bundles of cells that are heavily stained with fuchsin (white arrow). (B) Structures similar to those found in the pharynx are found throughout the ectoderm in the aboral part of the animal (white arrow). (C) The brightest concentration of staining occurs at the base of the mesenteries; (D) little to no staining was found along the tentacles. (E-E’) Uninjured animals appear to have less fluorescent labeling found throughout the animal when individuals are exposed to U0126. (F-H’) Time series of mucus staining during wound healing after head removal. (F-F’) One hour after injury fluorescent staining appears greatest near the wound epithelium. (G-G”) A brighter amount of staining appears present at four hours after injury, while U0126 animals show wound healing defects and less staining. (H-H’) By 12 hours, little mucin staining is visible in controls, where U0126 animals still exhibit wound healing defects, but appear to have elevated mucin levels. [file 1741-7007-12-24-S5.png]

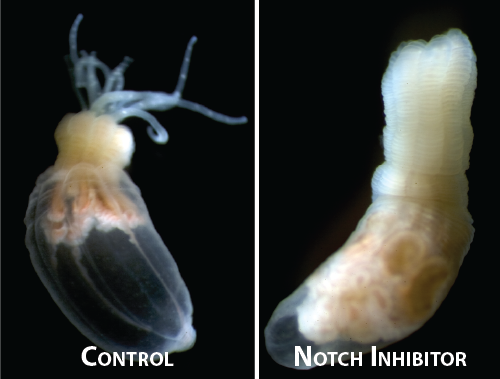

Supplement: Additional file 6 — Maintained apoptotic regulation during wound healing. (A) Quantification of apoptosis and nuclei count of five different stages of juvenile polyps before and after injury. Samples exhibit relatively equal numbers of TUNEL-labeled cells, where uninjured animals express the lowest number. (B) Ratio of apoptosis compared to total nuclei count shows maintained apoptosis throughout early puncture events. [file 1741-7007-12-24-S6.png]

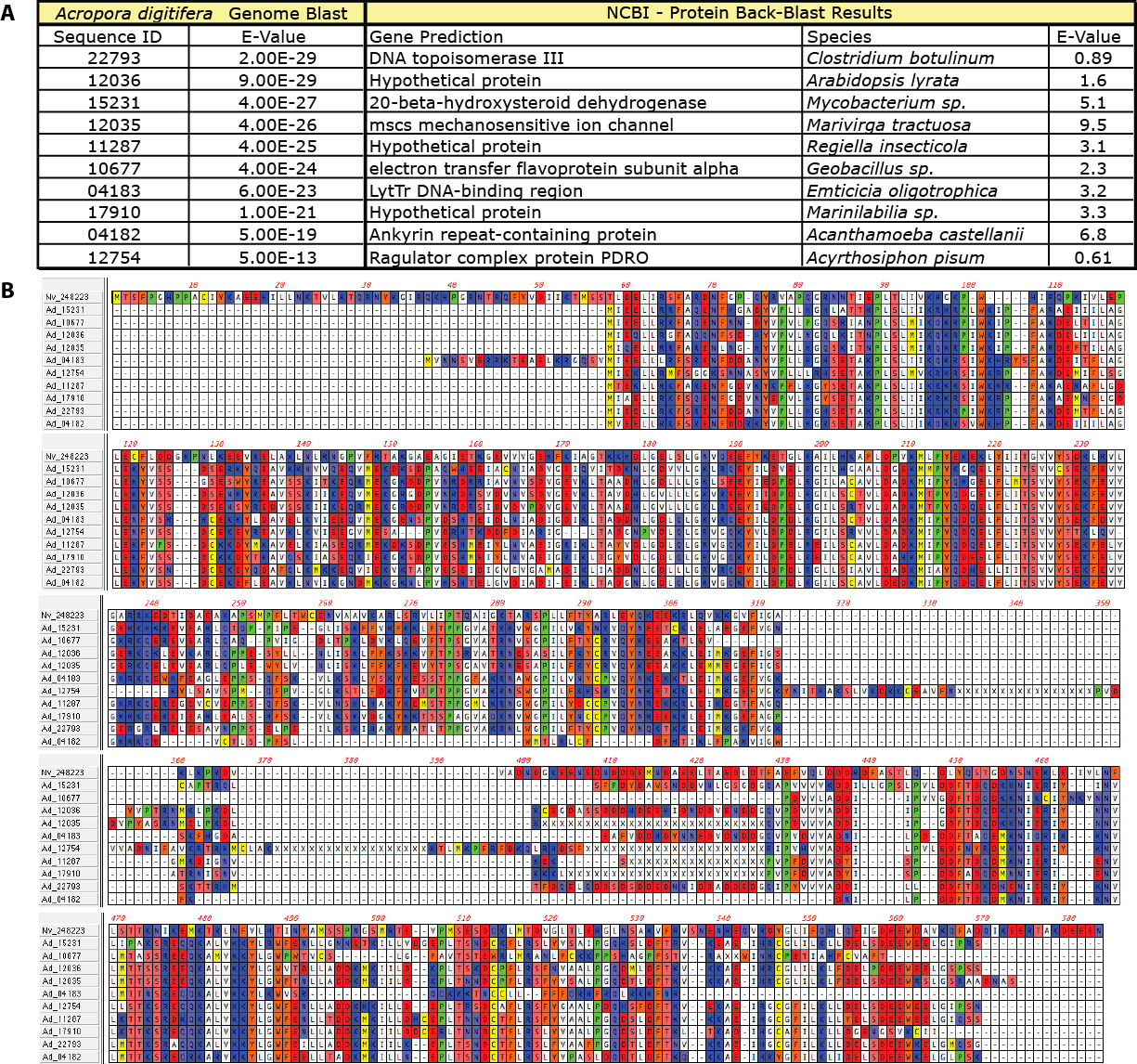

Supplement: Additional file 9 — The thiamine enzyme from Nematostella is likely a cnidarian-specific gene, derived from bacteria. This gene is found on scaffold 466 and spans positions 44637:52229. (A)Acropora digitifera genes closely related to the thiamine enzyme identified in Nematostella vectensis and their associated homology. (B) Alignment of Nematostella and Acropora sequences. [file 1741-7007-12-24-S9.png]

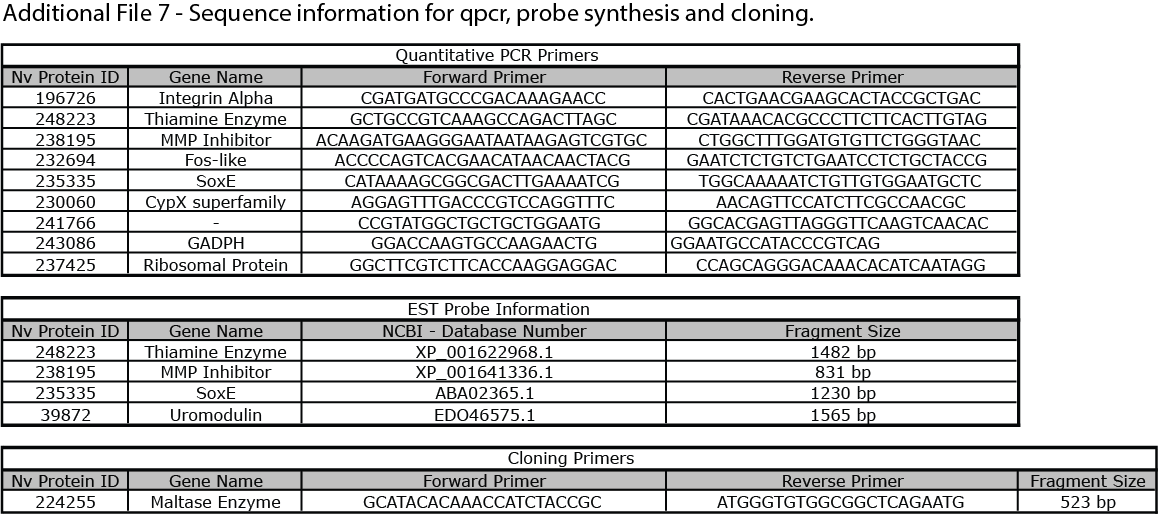

Supplement: Additional file 10 — Primer information for cloning. [file 1741-7007-12-24-S10.png]
